# Cerebral vasoreactivity in response to a head-of-bed position change is altered in patients with moderate and severe obstructive sleep apnea

**DOI:** 10.1371/journal.pone.0194204

**Published:** 2018-03-14

**Authors:** Clara Gregori-Pla, Gianluca Cotta, Igor Blanco, Peyman Zirak, Martina Giovannella, Anna Mola, Ana Fortuna, Turgut Durduran, Mercedes Mayos

**Affiliations:** 1 ICFO-Institut de Ciències Fotòniques, The Barcelona Institute of Science and Technology, Castelldefels (Barcelona), Spain; 2 Department of Respiratory Medicine, Hospital de la Santa Creu i Sant Pau, Barcelona, Spain; 3 Institució Catalana de Recerca i Estudis Avançats (ICREA), Barcelona, Spain; 4 CIBER Enfermedades Respiratorias (CibeRes) (CB06/06), Madrid, Spain; Technische Universitat Munchen, GERMANY

## Abstract

**Motivation:**

Obstructive sleep apnea (OSA) can impair cerebral vasoreactivity and is associated with an increased risk of cerebrovascular disease. Unfortunately, an easy-to-use, non-invasive, portable monitor of cerebral vasoreactivity does not exist. Therefore, we have evaluated the use of near-infrared diffuse correlation spectroscopy to measure the microvascular cerebral blood flow (CBF) response to a mild head-of-bed position change as a biomarker for the evaluation of cerebral vasoreactivity alteration due to chronic OSA. Furthermore, we have monitored the effect of two years of continuous positive airway pressure (CPAP) treatment on the cerebral vasoreactivity.

**Methodology:**

CBF was measured at different head-of-bed position changes (supine to 30° to supine) in sixty-eight patients with OSA grouped according to severity (forty moderate to severe, twenty-eight mild) and in fourteen control subjects without OSA. A subgroup (n = 13) with severe OSA was measured again after two years of CPAP treatment.

**Results:**

All patients and controls showed a similar CBF response after changing position from supine to 30° (p = 0.819), with a median (confidence interval) change of -17.5 (-10.3, -22.9)%. However, when being tilted back to the supine position, while the control group (p = 0.091) and the mild patients with OSA (p = 0.227) recovered to the initial baseline, patients with moderate and severe OSA did not recover to the baseline (9.8 (0.8, 12.9)%, p < 0.001) suggesting altered cerebral vasoreactivity. This alteration was correlated with OSA severity defined by the apnea-hypopnea index, and with mean nocturnal arterial oxygen saturation. The CBF response was normalized after two years of CPAP treatment upon follow-up measurements.

**Conclusion:**

In conclusion, microvascular CBF response to a head-of-bed challenge measured by diffuse correlation spectroscopy suggests that moderate and severe patients with OSA have altered cerebral vasoreactivity related to OSA severity. This may normalize after two years of CPAP treatment.

## Introduction

Obstructive sleep apnea (OSA) is a highly prevalent disorder associated with sleepiness [1, 2], neurocognitive impairment, cardiovascular and cerebrovascular disease [3–6], and increased morbidity and mortality [7, 8]. Strong evidence indicates that it is an independent risk factor for ischemic stroke (with a frequency in stroke of 30% to 80% [9–12]), including both longer time and poorer functional outcome from rehabilitation after ischemic stroke, and an increased risk of recurrence and mortality [13, 14]. The mechanisms responsible for those effects remain partly unknown. It is, however, known that repeated episodes of hypoxia-reoxygenation and increased sympathetic activity can activate different pathogenic pathways leading to oxidative stress, endothelial dysfunction, hypercoagulability and insulin resistance which promote atherogenesis [15–18]. OSA, hence, alters the basic control mechanisms for regulating cerebral blood flow (CBF) by decreasing resting CBF, impairing autoregulation and reducing cerebrovascular reserve capacity [19]. A blunted cerebrovascular reactivity, typically defined as the CBF change in response to a vasoactive stimulus such as hypercapnia, hypoxia or breath-hold has been described in patients with OSA [20–22]. An impaired compensatory response to cerebral hypoperfusion secondary to orthostatic hypotension has also been described [23]. These alterations to normal cerebrovascular control interfere with brain function and render the brain more vulnerable to ischemic events as brain tissue is particularly sensitive to hypoxic damage and rapid reperfusion.

There are several techniques for monitoring the state of cerebral vasoreactivity (CVR) in patients. However, many of these techniques are expensive, have limited availability and some are invasive and require exposure to ionizing radiation [24–28]. An optimal technique for hemodynamic monitoring which is non-invasive, portable, continuous, able to measure at the point-of-care or at the bed-side, and relatively inexpensive is still not present in the clinics. Transcranial Doppler ultrasound (TCD) fulfills many of these requirements. However, arteries cannot be insonated in part of the population, the estimation of the blood flow can be erroneous due to difficulties in estimating the artery diameter, and, finally, microvascular CVR could differ from the CVR of the macrovasculature measured by TCD, specially in pathological conditions [29]. Recently, diffuse correlation spectroscopy (DCS) has been proven to be a non-invasive transcranial optical method for measuring microvascular cortical CBF [24, 30]. DCS has been validated against other measures of CBF such as TCD [31, 32], arterial-spin labeled-magnetic resonance imaging [33–35] and xenon computed-tomography [36], and has been used to study the cerebral hemodynamic response derived from different stimulus such as acetazolamide injection [32], hypercapnia [22], visual and motor stimulation [30] and orthostatic stress [37–40] in a head-of-bed manipulation protocol.

The key result, relevant to this work, from the studies that measured CBF response to a mild orthostatic head-of-bed challenge [37–40] is that DCS is able to observe altered CVR in the brain cortex affected by different conditions such as ischemic stroke. We note that even though DCS studies are mainly limited to the frontal lobes, they have been shown to be a valid area of measurement when looking at the CVR with other techniques [41].

We have assumed that DCS allows us to assess microvascular CBF at bedside in a safe and well-tolerated manner and is suitable for studying CVR impairments of the frontal cortex in OSA syndrome. This allowed us to hypothesize that, compared with non-OSA subjects, patients with OSA would have an altered microvascular cerebral flow response to a mild orthostatic head-of-bed challenge. We have also hypothesized that this impairment is related to OSA severity.

Finally, we were motivated by the fact that some studies have detected an improvement of the impaired cerebrovascular reactivity response after treatment with continuous positive airway pressure (CPAP) [21, 42, 43]. This allowed us to hypothesize that the CVR impairment as measured by DCS on the frontal lobes could be ameliorated by long-term CPAP treatment.

For this purpose, we used DCS to study cerebral hemodynamic responses to a head-of-bed position change in a group of patients with OSA with different levels of severity, and compared their cerebral hemodynamics to a control group of healthy subjects. In addition, the effect of CPAP on brain hemodynamic changes was evaluated in a subgroup of patients with severe OSA after two years of CPAP treatment.

## Materials and methods

### Study design and participants

This study was conducted at a referral Sleep Unit (Department of Respiratory Medicine, Hospital de la Santa Creu i Sant Pau) in Barcelona, Spain. The study protocol was approved by the Ethical Committee of Clinical Investigation of Sanitary Health Management of the Hospital de la Santa Creu i Sant Pau (EC/11/001/1166). All participants gave their informed written consent. Two groups of subjects were enrolled: patients with OSA syndrome with an apnea-hypopnea index (AHI) ≥ 5, and healthy controls (AHI < 5). OSA severity was classified in two levels based on AHI (moderate and severe ≥ 15, and mild > 5 to 14.9 events/hour). Exclusion criteria were: being older than 80 years, receiving or having previously received CPAP treatment, presence of chronic obstructive pulmonary or neuromuscular diseases, previous ischemic stroke, and refusal to participate in the study. Anthropometric characteristics were obtained for all participants. A pre-established questionnaire was used to collect demographic variables including medical history, cardiovascular risk factors and current medication. Subjects were instructed to avoid caffeinated or alcoholic beverages during the hours prior to the study. Diagnosis of arterial hypertension (AHT) was established according to European Society of Hypertension/European Society of Cardiology criteria [44]. In a subgroup of patients with severe OSA (AHI > 30), the measurements were repeated after at least two years of CPAP treatment.

### Sleep studies

OSA diagnosis was performed by conventional full polysomnography (Siesta, Compumedics, Melbourne, Australia) or respiratory polygraphy (Embletta, Natus Medical, Middleton, USA) including, at least, the following parameters: oronasal flow (thermistor and nasal cannula), thoracoabdominal movements and pulse oximetry. The respiratory events included in the calculation of the AHI were defined according to the Spanish Sleep group [45] and the American Sleep Disorders Association guidelines [46]. The extent of self-reported sleepiness/drowsiness was analyzed using the Spanish version of the Epworth scale [47].

### Continuous positive airway pressure titration and compliance

All patients with severe OSA received CPAP treatment [45] after the study onset and part of these severe patients were measured with our protocol again after at least two years of treatment. CPAP titration was performed by an overnight polysomnography with manual CPAP titration or by autotitration devices [48]. Objective treatment compliance was determined by dividing the number of hours recorded by the CPAP device’s built-in hour meter by all the nights of the treatment period. Patients with an average use time of less than four hours per night were considered non-compliant.

### Optical methods and instrumentation

We have developed and applied a DCS system [24]. DCS uses long coherence length near-infrared light (785 nm) to probe the tissues [49]. On the tissue surface, it monitors the light intensity fluctuations of the speckle patterns in order to calculate an intensity autocorrelation function of this detected light. This intensity autocorrelation function is, then, fitted using the correlation diffusion equation in a semi-infinite geometry, obtaining a blood flow index of the local microvasculature [24, 49]. A custom probe consisting of a detector fibre set at 2.4 cm from a source fibre, providing information about the cerebral cortex hemodynamics, was placed centered on the right forehead avoiding the sinuses. We have assumed that cerebral hemodynamic changes due to orthostatic stress are global in patients without cerebrovascular disease [37]. The microvascular blood flow index was measured continuously with a three second temporal resolution during the study.

### Head-of-bed protocol

**Fig 1:**
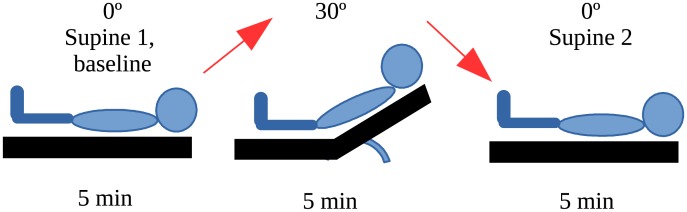
The protocol of the different head-of-bed position changes.

We have used a head-of-bed position change as a mild orthostatic challenge to induce CBF changes [37–39]. This protocol involved changing the head-of-bed angle from the supine baseline (0°) to 30° elevation and back to the initial supine position (0°). Fig 1 illustrates the protocol.

First, the subjects were asked to lie on a motorized movable bed to place and fix the optical probe on their foreheads. The protocol was started after about thirty minutes of preparation. During the experiment, the subjects were kept for five minutes in each head-of-bed position. The transitions between head-of-bed positions lasted a maximum of thirty seconds. The relative CBF (rCBF) changes for each position were obtained by normalizing the continuous blood flow index obtained for each position with the mean blood flow index corresponding to the initial supine position, and averaging the normalized values corresponding to each position. To avoid bed movement artifacts, the first minute and the last minute for each position were excluded in the analysis. rCBF response to the change from the supine position to 30° is represented as rCBF_supine to 30°_. rCBF response when tilting back to the initial supine position considering the baseline as the initial supine position is represented as rCBF_30° to supine_. All measurements were performed during the daytime.

### Statistical analysis

We have expressed quantitative variables as a median and interquartile range (median (Q1, Q3)), and categorical variables as number of cases and percentages (cases (percentages)). The Shapiro-Wilk test was used to assess for normality. The Kruskal-Wallis test (for quantitative dependent variables) or the Chi-squared test (for categorical dependent variables) were used with three-way pairwise comparisons with a Bonferroni correction. The Wilcoxon signed-rank test was used to assess the differences within the same group. The Wilcoxon rank-sum test (for quantitative variables) or the Chi-squared (for categorical variables) test were used to assess the differences between two groups. Spearman correlation was used to assess the correlation between quantitative variables. A multiple linear model (in a forward stepwise way) was used to linearly model the relationship between a dependent variable and the different quantitative and categorical variables. p < 0.05 was considered as the threshold for rejection of the null hypothesis for all statistical tests. All statistical analyses were performed with R [50] using the “PMCMR” [51] package.

## Results

### Baseline characteristics

A cohort of eighty-two (n = 82) subjects conformed the population of this study. Sixty-eight (n = 68) patients with OSA (forty moderate and severe, and twenty-eight mild) and fourteen control subjects were recruited. Full polysomnography was performed on thirty-two subjects (39%) and respiratory polygraphy on fifty subjects (61%).

Table 1 contains the demographics, clinical characteristics and sleep study results of the included subjects. Patients with moderate and severe OSA were older than the control group and more obese than both the patients with mild OSA and the controls. Gender distribution was different between groups, but pairwise comparisons with a Bonferroni correction did not provide evidence between which groups significant differences existed. Smoking, diabetes and dyslipidemia were similar among the three groups.

**Table 1:** a) Demographics, clinical characteristics and b) sleep study results.

**a)**					
	**Control (n = 14)**	**Mild (n = 28)**	**Moderate and severe (n = 40)**	**p**	**Total (n = 82)**
**Age (y.)**	52.5 (40, 56)	53.5 (47.5, 61)	57 (50, 62)	0.040*^a^	54 (48, 62)
**Males n (%)**	5 (36)	18 (64)	30 (75)	0.030*	53 (65)
**BMI (kg/cm^2^)**	24 (23, 26)	28 (25, 30)	31.5 (28, 35)	< 0.001*^b^	29 (25, 33)
**Epworth**	7 (5, 9)	12 (8, 15)	11 (7, 14.5)	0.040*^c^	10 (6, 14)
**AHT n (%)**	0 (0)	2 (7)	22 (55)	< 0.001*^b^	20 (24)
**Smoker n (%)**	5 (36)	17 (61)	18 (45)	0.325	49 (60)
**Diabetes n (%)**	0 (0)	3 (11)	9 (22.5)	0.094	11 (13)
**Dyslipidemia n (%)**	2 (14)	5 (18)	9 (22.5)	0.440	18 (22)
**b)**					
**AHI (n./hour)**	2 (1, 4)	9 (7, 12)	48.5 (21, 78)	< 0.001*^d^	14.5 (6, 47)
**Mean SpO_2_ (%)**	95.5 (95, 96)	95 (94, 96.5)	93 (92, 95)	< 0.001*^b^	94 (93, 96)
**CT90 (%)**	0 (0, 0)	0 (0, 0.3)	10 (3, 23)	< 0.001*^b^	0.4 (0, 11)
**ODI4 (%)**	1 (0, 3)	5 (3, 10)	44 (19, 71)	< 0.001*^b^	11 (3.5, 43)

Data shown as median (interquartile range) or number of cases (percentages).

Symbols indicate a statistically significant difference between the different groups (*), the moderate and severe group with OSA versus the control group (^a^), the moderate and severe group with OSA versus the other groups (^b^), the control group versus the mild group with OSA (^c^), and all the groups pairwise (^d^).

BMI, body mass index; AHT, arterial hypertension; AHI, apnea-hypopnea index; SpO_2_, arterial oxygen saturation; CT90, % of total sleep time with SpO_2_ lower than 90%; ODI4, 4% oxygen desaturation index; OSA, obstructive sleep apnea.

None of the control subjects were receiving chronic medications. Patients with OSA, on the other hand, were receiving chronic medications (the majority being anti-hypertensive); five received calcium channel blockers, eight angiotensin-converting enzyme inhibitor, six beta blockers, four diuretics, six angiotensin II receptor blockers, and two alopurinol.

Not all data (demographics, clinical characteristics, sleep study results and optical study results) was normally distributed. For consistency, we have opted to use statistical tests that are appropriate for non-normally distributed data.

### Orthostatic cerebral blood flow challenge: Patients versus the healthy control group

**Fig 2:**
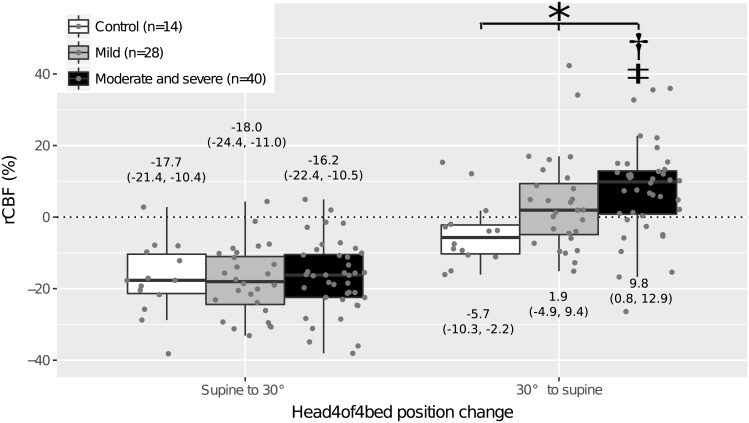
rCBF response to orthostatic stress induced by a mild head-of-bed position change for the different OSA severity groups (moderate and severe, and mild) and the control group. Measured mean data points for each patient and each head-of-bed position change, and classic boxplots for each group and each head-of-bed position change are shown. Labels show the median (interquartile range) for each group and each head-of-bed position change. Symbols indicate a statistically significant difference between the different groups (*), the group versus the control group (†), and the group versus the baseline (‡). rCBF, relative cerebral blood flow; OSA, obstructive sleep apnea.

As shown in Fig 2, the category of the patients assigned according to OSA severity was not a statistically significant factor for rCBF response to the change from the supine position to 30° (p = 0.819). In other words, all controls and patients showed a similar rCBF response after the first HOB position change. However, when being tilted back to the supine position, the category of the patients identified according to the OSA severity was a statistically significant factor (p = 0.004). The control group (p = 0.091) and the group with mild OSA (p = 0.227) recovered to the initial baseline while the group with moderate and severe OSA presented a statistically significantly higher blood flow relative to the initial supine position (p < 0.001). The recovery response of the group with moderate and severe OSA was significantly different from that of the control group (p = 0.003), though the recovery response of the group with mild OSA was not significantly different from that of the control group (p = 0.174).

### Association between sleep study results, demographics, clinical characteristics, and the cerebrovascular response

No statistically significant correlations were found between rCBF_supine to 30°_ and any of the sleep study results, demographics or clinical characteristics.

For all subjects measured, a positive statistically significant Spearman correlation (p = 0.007, see the supplementary material “S1 Table”) was found between rCBF_30° to supine_ and AHI, and a negative significant correlation was found between rCBF_30° to supine_ recovery and mean arterial oxygen saturation (SpO_2_) sleep night value (p = 0.002). Other positive relevant correlations were between rCBF_30° to supine_ and % of total sleep time with SpO_2_ lower than 90% (CT90) (p = 0.005), rCBF_30° to supine_ and 4% oxygen desaturation index (ODI4) (p = 0.048), and rCBF_30° to supine_ and body mass index (BMI) (p = 0.005). Age was not significantly correlated to rCBF_30° to supine_ (r_Spearman_ = 0.15, p = 0.199). Excluding the control group, only mean SpO_2_ was found to be correlated to rCBF_30° to supine_ (r_Spearman_ = -0.28, p = 0.023).

When performing a multiple linear model with rCBF_30° to supine_ as the dependent variable versus the different sleep study results, demographics and clinical characteristics as the independent variables, mean SpO_2_ was the independent variable whose inclusion gave the most statistically significant improvement of the fit with r_adj_^2^ (percentage of the response variable variation that is explained by a linear model) of 0.12 and a slope (*β*) of -2.02. No other secondary variables (AHI, natural logarithm of AHI, age, gender, smoking status, BMI, AHT, diabetes, dyslipidemia, CT90, ODI4 or Epworth scale) improved the model to a statistically significant extent. A second multiple linear model was performed excluding the control group. Again, a model with rCBF_30° to supine_ as the dependent variable and mean SpO_2_ as the independent variable was found with r_adj_^2^ of 0.09 and a slope (*β*) of -1.71.

A full subject-wise representation of the sleep study results, demographics, clinical characteristics, and optical study results can be found in the supplementary material “S2 Table”.

### Cerebral vasoreactivity in obstructive sleep apnea patients after long-term continuous positive airway pressure treatment

Thirteen (n = 13) patients with severe OSA were recruited again after 2.3 (2.0, 2.4) years of CPAP treatment. All the patients that were recruited again had good CPAP treatment compliance of 6.8 (5.3, 7.0) hours per night. Mean CPAP pressure was 10 (8-10) mmHg. Their first use of CPAP corresponded with the day after the first optical measurement. The same protocol (the HOB challenge) used in the first recruitment was performed again. The remaining patients treated with CPAP were excluded due to their refusal to participate in the second part of the study. None of the patients changed their condition with respect to diabetes, dyslipidemia or other relevant cardiovascular conditions (e.g. none had a stroke) during the CPAP treatment. The weight differences before the treatment and after two years were not statistically significant (p = 0.787) either. One patient changed from being non-hypertensive to being hypertensive together with the start of hypertensive treatment.

The supplementary table “S3 Table” contains the pre-treatment demographics, clinical characteristics, and the sleep study results of the subgroup of patients with severe OSA recruited after two years of CPAP treatment compared to the rest of the group with severe OSA measured one time, and to the complete severe OSA group. No statistically significant differences between demographics, clinical characteristics, or sleep study results were found. rCBF_30° to supine_ was not statistically significantly different between the patients with severe OSA remeasured and the patients measured one time (p = 0.053).

**Fig 3:**
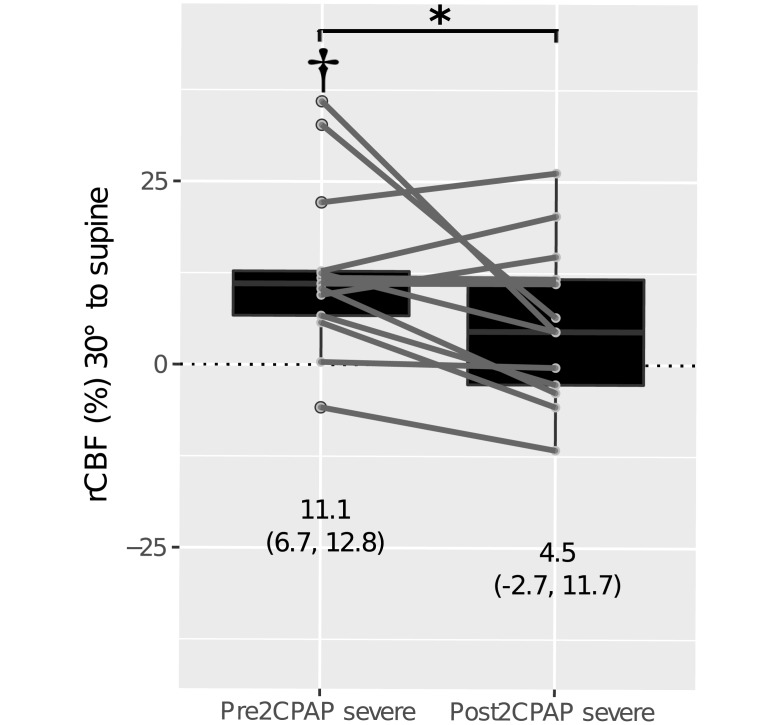
rCBF recovery after a head-of-bed position change and back to the supine position in patients with severe OSA before and after long-term CPAP treatment. Measured mean data points and classic boxplots are shown. Median (interquartile range) are shown in labels. Symbols indicate a statistically significant difference between the mean ranks (*), and from the baseline (†). rCBF, relative cerebral blood flow; CPAP, continuous positive airway pressure treatment.

The rCBF_supine to 30°_ for the group with severe OSA remeasured was again similar to two years before (p = 0.893). Fig 3 shows, in contrast, that after a head-of-bed change from the supine position to 30° and back to the supine position, the magnitude of rCBF_30° to supine_ changed significantly from the pre- to post-CPAP period (p = 0.047). The pre-CPAP group with severe OSA did not recover to the baseline (p = 0.001), while the post-CPAP group with severe OSA did (p = 0.094).

A full subject-wise representation of the demographics, sleep study results, clinical characteristics and optical study results of the severe patients measured before and after CPAP treatment can be found in the supplementary material “S4 Table”.

## Discussion

To the best of our knowledge this is the first study that evaluates the cerebral microcirculatory vasoreactivity in response to a mild orthostatic stress in patients with OSA. The main finding is the different response of the microvascular blood flow in patients with moderate and severe OSA as compared to patients with mild OSA and healthy control subjects. In patients with moderate and severe OSA the orthostatic challenge leads to an alteration of the supine CBF upon returning to the initial HOB supine position. We found that this alteration is related to OSA severity, AHI and mean SpO_2_, and that long-term CPAP treatment ameliorates this alteration.

In our study with DCS, the amount of CBF change in response to a HOB from the supine position to 30° was similar for all subjects measured, and in agreement with previous DCS studies. Edlow et al. [37], in healthy patients, found a response of 18 (± 1.5)% (mean(standard error)) cerebral blood flow. In ischemic stroke patients in a similar protocol, Durduran et al. [39] obtained a change of 30 (±7)% and 25 (±7)% in the ipsi- and contra-infarct hemispheres respectively, and Favilla et al. [38] applying the same protocol, found a change of 17 (±4.6)% and 15 (±4.6)% in the ipsi- and contra-infarct hemispheres, respectively. Considering these studies with control subjects, stroke patients and the results presented in this study including patients with OSA, a HOB change from the supine position to 30° may be a too mild challenge to be able to observe differences in the CVR of the brains of this population.

Only Edlow et al. [37], in healthy patients, evaluated the tilting back to the baseline level finding a significant increase of 9.7 (±1.8)% in sixty patients. In our study rCBF_30° to supine_ was different between cohorts grouped by OSA severity. Patients with moderate and severe OSA presented a significantly higher blood flow than the corresponding value at the initial supine position, while the controls and the patients with mild OSA did not. This result suggests that patients with OSA might need a longer time period than five minutes in each HOB position to stabilize their hemodynamic response. This is assuming that healthy cerebral auto-regulation would, given enough time, normalize the CBF. Urbano et al. [23] measured (by TCD) the effect of a strong orthostatic challenge (standing to squatting position) in twenty-six patients with moderate or severe OSA and twenty-eight control subjects, and showed that patients with moderate or severe OSA presented an impaired compensatory response to a strong orthostatic challenge. While the degree of change was similar between the two groups, patients with OSA had a significantly slower rate of recovery of blood pressure, cerebral blood flow velocity and cerebro-vascular conductance than the control group. However, this study did not consider the correlation between OSA severity (AHI) and the recovery time. Even though there are differences in design and protocol, the results are in accordance with our findings, showing that patients with OSA might take a longer time than healthy subjects to recover cerebral blood flow to baseline after an orthostatic stress. This hypothesis needs to be checked in future studies in which patients should stay for longer periods of time in each HOB position. Unfortunately, this is a non-trivial set-up because of potential confounders since patients often do not comply well with protocols including long resting periods.

Other studies have looked at the CVR in patients with OSA with conflicting results. Busch et al. [22], who also used DCS, found that children with OSA and snorers have a blunted CBF response to hypercapnia during wakefulness compared to a control group. Placidi et al. [52] used a breath-holding test finding a reduction of CVR in a group of patients with severe OSA when compared to controls. In contrast to these two studies, Foster et al. [53] and Ryan et al. [54] did not detect differences in a small group of patients using other testing procedures for hypercapnia induced stimulation. Reichmuth et al. [42] studied twenty patients with moderate and severe OSA and twenty controls, showing that cerebral vasodilation response to hypoxia and hypercapnia was smaller in patients versus controls. They also found that hypoxia induced vasodilation in the forearm, though not vasodilatation induced by hypercapnia, was attenuated in patients with OSA. Morgan et al. [20] described a blunted cerebrovascular response to hyperoxic hypercapnia in a large sample of 373 participants from the Sleep Heart Health Study cohort. They observed a positive correlation between the mean level of arterial oxygen during sleep and vascular cerebral responsiveness to hypercapnia. In contrast, AHI was not statistically significantly associated with cerebrovascular carbon dioxide reactivity. Nasr et al. [55] evaluated cerebral autoregulation (the arterial blood pressure-cerebral blood flow relationship) in patients with moderate OSA and age-matched control subjects. Cerebral autoregulation was impaired in patients with OSA during wakefulness with a strong relationship between the impairment and the number of apneas and hypopneas during sleep, suggesting that patients with the most severe OSA had the strongest alteration of cerebral autoregulation.

We have found significant correlations with rCBF_30° to supine_ recovery and with both AHI and mean SpO_2_, though mean SpO_2_ was the best predictor when performing a multiple linear model. Other tested predictors that did not improve this model were age, gender, smoking status, BMI, AHT, diabetes, dyslipidemia, CT90, ODI4 and Epworth scale. When considering only patients with OSA, a significant correlation with rCBF_30° to supine_ recovery and mean SpO_2_ was found again, and mean SpO_2_ was the best predictor in the multiple linear model again. This result is in agreement with the hypothesis that the value of nocturnal saturation over the number of apneas and hypopneas better predicts the degree of endothelial impairment, which is one of the main factors involved in cerebral dysfunction in OSA [19]. In line with our results, other authors have described a significant correlation between cerebrovascular reactivity and nocturnal hypoxemia [21, 43].

Impaired CVR may imply impaired cerebral autoregulation, which, in turn, may contribute to the increased risk of stroke in patients with OSA through two physiopathological pathways: an increased vulnerability to drops in arterial blood pressure leading to ischemia in the brain, and, an excess of flow in vessels in the brain during surges in arterial blood pressure leading to capillary damage [56]. In addition, impaired cerebral autoregulation might contribute to the poorer neurological outcome that has been reported in stroke patients with associated OSA [13].

After at least two years of CPAP treatment, thirteen patients with severe OSA were measured again and CVR was shown to have normalized. In general, no significant clinical parameters nor medication were changed during this period. Even though only a small group of patients was measured again, other studies support a recovery of CVR due to CPAP treatment. Reichmuth et al. [42] found that hypoxic vasodilation improved after twelve months of CPAP treatment. Prilipko et al. [43] in twenty-three patients with moderate or severe OSA using a breath-holding stimulus and functional magnetic resonance imaging, found that the CVR of the thalamus was increased after two months of CPAP treatment, while it decreased in patients with sham CPAP. Foster et al. [21] recruited eight patients with severe OSA who went through isocapnic hypoxia. Cerebral blood flow velocity response was significantly lower in patients than in control subjects, though the response was similar between patients with OSA and controls after four-to-six weeks of CPAP treatment. Taken together, these results support the claim that OSA related impairment in cerebrovascular function is reversible, at least partly, with treatment.

Our study has some potential limitations that should be taken in consideration. First, measurements were performed at different times during hospital working hours (74% of the patients in the afternoon) without considering the fact that it has been suggested that CVR diminishes in the morning in patients with OSA after the continuous stress of night hypoxemia and hypercapnia. In line with this, Placidi et al. [52] found significantly lower values of breath-holding index in the morning than in the afternoon in patients with OSA by TCD. However, considering that the majority of moderate and severe subjects (70%) were measured in the afternoon, this factor is not expected to affect our results. This important aspect should be monitored in future studies.

Second, the study included patients with different OSA severity and control subjects that were not completely matched in demographics and clinical characteristics. In particular, BMI and AHT were significantly higher in patients with moderate and severe OSA. Chronic hypertension is involved independently in the production of reactive oxygen species, inflammation, endothelial dysfunction and vasodilatation impairment; therefore, the association of OSA, with a frequency in resistant hypertension of more than 80% [57], and hypertension could have an accumulative effect [58]. Considering that 91% of AHT subjects were patients with moderate and severe OSA, it is difficult to discriminate this effect in our dataset. Even though differences on age were found between OSA severity groups, Edlow et al. [37] found no difference in rCBF_30° to supine_ depending on age. In addition, while gender effect is not clear in our results, Edlow et al. found it to be a significant effect. However, relevantly, when performing the multiple linear model for rCBF_30° to supine_ as the dependent variable versus the different sleep study results, demographics, and clinical characteristics, only mean SpO_2_ was identified as the best significant factor. Neither AHT, BMI, age nor gender improved the model. As mentioned previously, the same results were found when the analysis was only performed in patients with OSA.

Third, the partial pressure of carbon dioxide (PCO_2_) was an unexplored relevant factor since the majority of the PCO_2_ baseline values for the subjects included were not obtained. PCO_2_ was not monitored during the orthostatic challenge. In any case, the literature shows that while hypoxemia is a factor that clearly modifies the cerebral autoregulation in patients with OSA, the results of the few studies that analyze cerebrovascular response to hypercapnia are contradictory [20, 23, 42]. The role of hypercapnia in the cerebrovascular response in patients with OSA needs to be investigated further in future studies.

Fourth, near-infrared diffuse correlation spectroscopy technique measures information from the brain, but also contains scalp and skull contributions [59–61]. However, when fitting only the early portion of the intensity autocorrelation function, our brain-to-scalp sensitivity increased from 25% to 45% approximately. This brain-to-scalp sensitivity is greater than the 10% sensitivity found by near-infrared spectroscopy technique [62]. Overall, even though our measurements contain extracerebral contributions, we can say that a relevant contribution is due to the brain as shown by other earlier studies [30, 62, 63].

Fifth, the use of respiratory polygraphy may underestimate the apnea or hypopnea index since the analysis does not distinguish between the awake and sleep periods, while polysomnography considers only the sleep periods. Moreover, respiratory polygraphy does not include respiratory events that lead to arousal without a significant desaturation of the arterial blood. This underestimation would not affect the patients currently labeled as being with severe OSA; instead, it would affect patients listed as moderate who should have been listed as being severe. However, since we have considered patients with moderate and severe OSA together for the analysis, this underestimation will not affect our main findings.

Finally, when looking at the CPAP effect, only a small group of patients, though with the strength of an excellent CPAP compliance, were reviewed. These results need to be confirmed in the near future in a large and clinically representative sample of patients with OSA.

### Conclusions

In summary, this study shows an alteration of the cerebrovascular reactivity at the level of the microvasculature in response to a mild orthostatic challenge in patients with moderate and severe OSA. This impairment, which is dependent on OSA severity, is an important pathogenetic link between OSA and vascular disease. The alteration in vascular regulation could negatively impact tissue perfusion during acute episodes of apnea, and indicate a potential greater vulnerability of the cerebral vasculature to stroke injury. For this study we have used a non-invasive technique, DCS, which enables continuous bedside monitoring of CBF and, a mild orthostatic head-of-bed challenge. The orthostatic stress stimulus mimics a daily action without the need for pharmacological agents, hypoxemia or hypercapnia exposures, and with minimal patient collaboration. We propose that this stimulus together with diffuse correlation spectroscopy can be a relatively simple and easy protocol for identifying subclinical alterations of cerebrovascular reactivity. Finally, we found that cerebral vasoreactivity improved with CPAP treatment in a small group of highly compliant patients, suggesting that the impairment in cerebrovascular function may be reversible, at least in part, with treatment. Further studies will be needed to better define the importance of this finding.

## Supporting information

S1 Table Correlations with optical study results, sleep study results and demographics of all subjects measured. Spearman correlations shown. * indicates a statistically significant correlation. rCBF, relative cerebral blood flow; SpO_2_, arterial oxygen saturation; AHI, apnea-hypopnea index; ODI4, 4% oxygen desaturation index; CT90, % of total sleep time with SpO_2_ lower than 90%; BMI, body mass index.
(PDF)

S2 Table Sleep study results, demographics, clinical characteristics, and optical study results of every patient measured. AHI, apnea-hypopnea index; SpO_2_, arterial oxygen saturation; ODI4, 4% oxygen desaturation index; CT90, % of total sleep time with SpO_2_ lower than 90%; AHT, arterial hypertension; DM, diabetes mellitus; DLP, dyslipidemia; rCBF, relative cerebral blood flow.
(PDF)

S3 Table Pre-CPAP treatment a) demographics, clinical characteristics and b) sleep study results of the subgroup with severe OSA remeasured after two years of CPAP treatment, the subgroup with severe OSA measured one time, and all the group with severe OSA. Data shown as median (interquartile range) or number of cases (percentages). BMI, body mass index; AHT, arterial hypertension; AHI, apnea-hypopnea index; SpO_2_, arterial oxygen saturation; CT90, % of total sleep time with SpO_2_ lower than 90%; ODI4, 4% oxygen desaturation index; OSA, obstructive sleep apnea; CPAP, continuous positive airway pressure treatment.
(PDF)

S4 Table Demographics, sleep study, clinical characteristics, results and optical study results for the severe obstructive sleep apnea patients measured before and after CPAP treatment. AHI, apnea-hypopnea index; SpO_2_, arterial oxygen saturation; ODI4, 4% oxygen desaturation index; CT90, % of total sleep time with SpO_2_ lower than 90%; CPAP, continuous positive airway pressure; AHT, arterial hypertension; DM, diabetes mellitus; DLP, dyslipidemia; rCBF, relative cerebral blood flow.
(PDF)
