# Supplementary material for: Cerebral vasoreactivity in response to a head-of-bed position change is altered in patients with moderate and severe obstructive sleep apnea
Source: PLoS One. 2018 Mar 14;13(3):e0194204. doi: 10.1371/journal.pone.0194204 (PMC5851619; doi:10.1371/journal.pone.0194204)
Supplement: S1 Table — Spearman correlations shown. * indicates a statistically significant correlation. rCBF, relative cerebral blood flow; SpO2, arterial oxygen saturation; AHI, apnea-hypopnea index; ODI4, 4% oxygen desaturation index; CT90, % of total sleep time with SpO2 lower than 90%; BMI, body mass index. (PDF) [file pone.0194204.s001.pdf]

S1 Table: Correlations with optical study results, sleep study results and demographics of all subjects measured.

**$r_{Spearman}$ (p)**	**$rCBF_{30° \text{ to supine}}$ (%)**	**Mean SpO_2 (%)**	**AHI (n/hour)**	**ODI4 (%)**	**CT90 (%)**
**Mean SpO_2 (%)**	-0.34 (0.002)*				
**AHI (n/hour)**	0.30 (0.007)*	-0.58 (<0.001)*			
**ODI4 (%)**	0.22 (0.048)*	-0.57 (<0.001)*	0.92 (<0.001)*		
**CT90 (%)**	0.31 (0.005)*	-0.76 (<0.001)*	0.85 (<0.001)*	0.81 (<0.001)*	
**BMI (kg/cm^2)**	0.31 (0.005)*	-0.53 (<0.001)*	0.57 (<0.001)*	0.56 (<0.001)*	0.59 (<0.001)*

Spearman correlations shown.* indicates a statistically significant correlation.

rCBF, relative cerebral blood flow; SpO_2, arterial oxygen saturation by pulse oximetry; AHI, apnea-hypopnea index; ODI4, 4% oxygen desaturation index; CT90, % of total sleep time with SpO_2 lower than 90%; BMI, body mass index.
