# Supplementary material for: Cerebral vasoreactivity in response to a head-of-bed position change is altered in patients with moderate and severe obstructive sleep apnea
Source: PLoS One. 2018 Mar 14;13(3):e0194204. doi: 10.1371/journal.pone.0194204 (PMC5851619; doi:10.1371/journal.pone.0194204)
Supplement: S2 Table — AHI, apnea-hypopnea index; SpO2, arterial oxygen saturation; ODI4, 4% oxygen desaturation index; CT90, % of total sleep time with SpO2 lower than 90%; AHT, arterial hypertension; DM, diabetes mellitus; DLP, dyslipidemia; rCBF, relative cerebral blood flow. (PDF) [file pone.0194204.s002.pdf]

S2 Table: Sleep study results, demographics, clinical characteristics, and optical study results of every patient measured.

ID	Positional change	Patient type	AHI (n./hour)	Mean SpO_2 (%)	ODI4 (%)	CT90 (%)	Male (yes=0)	Age (y.)	Weight (Kg)	Height (m)	Smoking (current/exsmoker=1)	AHT (yes=1)	DM (yes=1)	DLP (yes=1)	Epworth	rCBF (%)
2	0º to 30º	severe	74.8	92	63	19.3	0	65	88.6	1.65	1	1	0	0	8	-21.03
2	30º to 0º	severe	74.8	92	63	19.3	0	65	88.6	1.65	1	1	0	0	8	-9.62
3	0º to 30º	severe	99.5	92	73.7	22.8	1	63	94	1.59	0	1	1	0	15	-24.64
3	30º to 0º	severe	99.5	92	73.7	22.8	1	63	94	1.59	0	1	1	0	15	32.74
4	0º to 30º	severe	82.5	91	75.8	25.8	0	54	87.6	1.65	1	1	0	0	14	-15.58
4	30º to 0º	severe	82.5	91	75.8	25.8	0	54	87.6	1.65	1	1	0	0	14	-4.92
5	0º to 30º	severe	74.2	94	78.4	10.8	0	54	86.2	1.76	1	1	0	0	7	-21.3
5	30º to 0º	severe	74.2	94	78.4	10.8	0	54	86.2	1.76	1	1	0	0	7	0.36
6	0º to 30º	severe	72.8	89	68.9	38	0	73	90	1.61	0	1	0	0	6	4.93
6	30º to 0º	severe	72.8	89	68.9	38	0	73	90	1.61	0	1	0	0	6	9.52
8	0º to 30º	severe	91.1	86	88	58.6	0	49	152	1.81	1	0	0	0	23	-10.59
8	30º to 0º	severe	91.1	86	88	58.6	0	49	152	1.81	1	0	0	0	23	10.39
9	0º to 30º	severe	96.2	90	85.3	41.7	1	47	79.4	1.6	0	0	0	0	20	-7.64
9	30º to 0º	severe	96.2	90	85.3	41.7	1	47	79.4	1.6	0	0	0	0	20	11.85
11	0º to 30º	severe	79.8	92	66.2	23.1	0	69	100	1.73	1	0	1	1	9	-16.17
11	30º to 0º	severe	79.8	92	66.2	23.1	0	69	100	1.73	1	0	1	1	9	12.47
13	0º to 30º	severe	78.1	93	72.4	16.9	0	58	114	1.81	1	1	0	0	9	-28.33
13	30º to 0º	severe	78.1	93	72.4	16.9	0	58	114	1.81	1	1	0	0	9	6.71
14	0º to 30º	severe	97.5	95	96.3	14	0	48	110	1.79	1	1	0	0	10	-28.5
14	30º to 0º	severe	97.5	95	96.3	14	0	48	110	1.79	1	1	0	0	10	35.57
15	0º to 30º	severe	93.7	91	31	27.4	1	67	118	1.62	1	1	0	1	2	-24.74
15	30º to 0º	severe	93.7	91	31	27.4	1	67	118	1.62	1	1	0	1	2	7.55

16	0º to 30º	severe	36.1	93	36.4	14	0	62	96.6	1.71	0	1	1	0	13	-9.1
16	30º to 0º	severe	36.1	93	36.4	14	0	62	96.6	1.71	0	1	1	0	13	4.81
19	0º to 30º	severe	77.4	93	73.8	26	0	54	137	1.71	1	1	1	1	2	1.98
19	30º to 0º	severe	77.4	93	73.8	26	0	54	137	1.71	1	1	1	1	2	14.9
20	0º to 30º	severe	93.7	94	85.5	8.6	0	62	108	1.69	1	0	1	0	10	-34.9
20	30º to 0º	severe	93.7	94	85.5	8.6	0	62	108	1.69	1	0	1	0	10	-16.73
25	0º to 30º	severe	46.1	96	45.9	5.2	0	57	89	1.65	1	1	0	0	15	-16.25
25	30º to 0º	severe	46.1	96	45.9	5.2	0	57	89	1.65	1	1	0	0	15	22.15
26	0º to 30º	severe	55	94	54.2	3.8	0	53	125	1.74	1	1	0	0	14	-16.39
26	30º to 0º	severe	55	94	54.2	3.8	0	53	125	1.74	1	1	0	0	14	-0.76
27	0º to 30º	severe	71.2	86	70.5	58.3	0	53	89	1.58	1	1	0	1	23	-2.85
27	30º to 0º	severe	71.2	86	70.5	58.3	0	53	89	1.58	1	1	0	1	23	35.96
28	0º to 30º	severe	50	94	50.4	10	0	47	96	1.84	1	0	0	0	16	-1.45
28	30º to 0º	severe	50	94	50.4	10	0	47	96	1.84	1	0	0	0	16	11.25
29	0º to 30º	severe	77	93	55.4	15.5	1	64	72	1.56	0	0	0	0	12	-18.83
29	30º to 0º	severe	77	93	55.4	15.5	1	64	72	1.56	0	0	0	0	12	-5.32
30	0º to 30º	severe	34.2	91	35.4	12.7	0	57	98.2	1.78	1	0	1	1	15	-15.64
30	30º to 0º	severe	34.2	91	35.4	12.7	0	57	98.2	1.78	1	0	1	1	15	12.76
32	0º to 30º	severe	35	92	17.8	3.4	0	62	85.6	1.65	1	1	0	0	2	-11.17
32	30º to 0º	severe	35	92	17.8	3.4	0	62	85.6	1.65	1	1	0	0	2	15.4
33	0º to 30º	severe	47	94	42.6	8.6	0	47	82	1.79	0	0	0	0	11	-21.09
33	30º to 0º	severe	47	94	42.6	8.6	0	47	82	1.79	0	0	0	0	11	5.75
35	0º to 30º	severe	43	93	39	12	0	58	87	1.8	1	0	0	0	15	-22.48
35	30º to 0º	severe	43	93	39	12	0	58	87	1.8	1	0	0	0	15	-2.62
37	0º to 30º	severe	33	94	19.1	0	0	73	82.6	1.6	1	1	1	1	3	-8.73
37	30º to 0º	severe	33	94	19.1	0	0	73	82.6	1.6	1	1	1	1	3	7.35
45	0º to 30º	severe	33.8	95	19.3	0.8	1	64	66	1.6	0	0	0	0	6	-31.14

45	30º to 0º	severe	33.8	95	19.3	0.8	1	64	66	1.6	0	0	0	0	6	-5.83
47	0º to 30º	severe	71.5	91	64	31.4	0	61	118	1.66	1	1	1	1	14	-1.73
47	30º to 0º	severe	71.5	91	64	31.4	0	61	118	1.66	1	1	1	1	14	11.06
63	0º to 30º	severe	92.1	90	92.6	43.3	0	47	93.2	1.72	1	0	0	0	22	-36.02
63	30º to 0º	severe	92.1	90	92.6	43.3	0	47	93.2	1.72	1	0	0	0	22	-15.39
77	0º to 30º	severe	69	93	59.6	21	1	61	75	1.6	0	0	0	0	11	-15.29
77	30º to 0º	severe	69	93	59.6	21	1	61	75	1.6	0	0	0	0	11	2.12
17	0º to 30º	moderate	19.5	93	21.8	3	0	66	91	1.7	0	0	0	0	2	-22.43
17	30º to 0º	moderate	19.5	93	21.8	3	0	66	91	1.7	0	0	0	0	2	1.28
21	0º to 30º	moderate	20	94	8.2	2	0	61	98	1.69	1	0	0	0	7	-15.5
21	30º to 0º	moderate	20	94	8.2	2	0	61	98	1.69	1	0	0	0	7	15.01
22	0º to 30º	moderate	24	95	24.7	7	0	52	61	1.57	0	0	0	0	2	-16.06
22	30º to 0º	moderate	24	95	24.7	7	0	52	61	1.57	0	0	0	0	2	10.66
31	0º to 30º	moderate	19	93	13.5	4	1	50	71	1.56	1	0	0	0	NA	-18.27
31	30º to 0º	moderate	19	93	13.5	4	1	50	71	1.56	1	0	0	0	NA	10.17
38	0º to 30º	moderate	19	96	17	0.1	1	55	75	1.58	0	0	0	0	12	-19.74
38	30º to 0º	moderate	19	96	17	0.1	1	55	75	1.58	0	0	0	0	12	6.17
39	0º to 30º	moderate	19.4	94	4.1	0	1	62	65	1.58	0	0	0	0	10	-23.05
39	30º to 0º	moderate	19.4	94	4.1	0	1	62	65	1.58	0	0	0	0	10	11.7
41	0º to 30º	moderate	17.8	96	18.2	0.2	1	48	167	1.74	1	1	0	0	7	-13.39
41	30º to 0º	moderate	17.8	96	18.2	0.2	1	48	167	1.74	1	1	0	0	7	1
42	0º to 30º	moderate	17.2	96	8.8	0	0	69	81	1.79	1	0	1	1	10	-18.47
42	30º to 0º	moderate	17.2	96	8.8	0	0	69	81	1.79	1	0	1	1	10	12.04
44	0º to 30º	moderate	17	95	10.4	0.7	0	44	75	1.69	1	0	0	0	15	-10.72
44	30º to 0º	moderate	17	95	10.4	0.7	0	44	75	1.69	1	0	0	0	15	22.69
48	0º to 30º	moderate	15.8	95	17.1	2.2	0	53	76	1.74	1	1	0	0	3	-10.07

48	30º to 0º	moderate	15.8	95	17.1	2.2	0	53	76	1.74	1	1	0	0	3	13.31
56	0º to 30º	moderate	19	96	7	0.5	0	44	74	1.7	1	0	0	1	12	-7.18
56	30º to 0º	moderate	19	96	7	0.5	0	44	74	1.7	1	0	0	1	12	19.41
75	0º to 30º	moderate	21	96	19	5.4	0	34	73	1.74	0	0	0	0	12	-38.08
75	30º to 0º	moderate	21	96	19	5.4	0	34	73	1.74	0	0	0	0	12	-26.4
10	0º to 30º	mild	14.3	96	0	0	0	52	65.8	1.71	1	1	0	0	17	-7.55
10	30º to 0º	mild	14.3	96	0	0	0	52	65.8	1.71	1	1	0	0	17	4.98
23	0º to 30º	mild	8	94	5.1	0.3	0	76	74	1.71	1	0	1	1	2	-20.12
23	30º to 0º	mild	8	94	5.1	0.3	0	76	74	1.71	1	0	1	1	2	-9.36
24	0º to 30º	mild	10.9	95	11.2	2.6	0	69	81.1	1.71	1	1	1	0	9	-30.71
24	30º to 0º	mild	10.9	95	11.2	2.6	0	69	81.1	1.71	1	1	1	0	9	1.91
36	0º to 30º	mild	9.4	96	9.3	0	0	50	75	1.85	1	0	0	0	10	-33.13
36	30º to 0º	mild	9.4	96	9.3	0	0	50	75	1.85	1	0	0	0	10	-9.57
40	0º to 30º	mild	13.8	93	13.6	0.3	0	58	91.8	1.7	1	0	0	1	12	-15.65
40	30º to 0º	mild	13.8	93	13.6	0.3	0	58	91.8	1.7	1	0	0	1	12	34.11
46	0º to 30º	mild	14	91	8.5	10	1	53	94	1.69	1	0	0	0	11	-30.47
46	30º to 0º	mild	14	91	8.5	10	1	53	94	1.69	1	0	0	0	11	42.32
50	0º to 30º	mild	12	94	4	0.2	0	49	92	1.79	1	0	0	0	14	-26.11
50	30º to 0º	mild	12	94	4	0.2	0	49	92	1.79	1	0	0	0	14	16.83
51	0º to 30º	mild	5	96	2.9	0	0	43	125	1.74	1	0	0	0	14	-9.9
51	30º to 0º	mild	5	96	2.9	0	0	43	125	1.74	1	0	0	0	14	4.17
52	0º to 30º	mild	14.7	97	13.7	0	0	51	87.4	1.75	0	0	0	0	6	-29.52
52	30º to 0º	mild	14.7	97	13.7	0	0	51	87.4	1.75	0	0	0	0	6	-15.12
53	0º to 30º	mild	13.3	97	13.3	0	1	51	65.6	1.63	0	0	1	0	12	-13.22
53	30º to 0º	mild	13.3	97	13.3	0	1	51	65.6	1.63	0	0	1	0	12	-7.33
54	0º to 30º	mild	6	94	6.6	0	1	51	50	1.64	1	0	0	1	8	-17.48
54	30º to 0º	mild	6	94	4	0	1	51	50	1.64	1	0	0	1	8	0.13

55	0º to 30º	mild	10.4	97	7.7	0	1	69	74	1.62	0	0	0	0	6	-13.95
55	30º to 0º	mild	10.4	97	7.7	0	1	69	74	1.62	0	0	0	0	6	7.94
57	0º to 30º	mild	9	99	0.2	0	1	54	58	1.4	0	0	0	0	16	-15.87
57	30º to 0º	mild	9	99	0.2	0	1	54	58	1.4	0	0	0	0	16	1.96
58	0º to 30º	mild	5.5	98	0.9	0	0	41	73	1.64	1	0	0	0	20	-20.54
58	30º to 0º	mild	5.5	98	0.9	0	0	41	73	1.64	1	0	0	0	20	-12.77
59	0º to 30º	mild	9.3	94	1.9	0.2	1	59	83.4	1.61	1	0	0	0	17	-8.1
59	30º to 0º	mild	9.3	94	1.9	0.2	1	59	83.4	1.61	1	0	0	0	17	16.99
60	0º to 30º	mild	14	92	4	15	0	69	108	1.69	1	0	0	0	10	-29.31
60	30º to 0º	mild	14	92	4	15	0	69	108	1.69	1	0	0	0	10	-4.55
61	0º to 30º	mild	5	95	5.1	0	0	72	83	1.65	0	0	0	0	8	-1.19
61	30º to 0º	mild	5	95	5.1	0	0	72	83	1.65	0	0	0	0	8	8.84
62	0º to 30º	mild	12.5	95	16	0.4	0	38	98	1.63	1	0	0	0	8	-19
62	30º to 0º	mild	12.5	95	16	0.4	0	38	98	1.63	1	0	0	0	8	1.26
65	0º to 30º	mild	6	96	5.7	0	0	41	68	1.66	0	0	0	0	14	-21.54
65	30º to 0º	mild	6	96	5.7	0	0	41	68	1.66	0	0	0	0	14	-4
67	0º to 30º	mild	9.7	94	1	3.2	1	68	71	1.63	0	0	0	1	13	-23.12
67	30º to 0º	mild	9.7	94	1	3.2	1	68	71	1.63	0	0	0	1	13	16.07
68	0º to 30º	mild	6	94	5.1	0	0	31	74	1.57	1	0	0	0	2	-17.45
68	30º to 0º	mild	6	94	5.1	0	0	31	74	1.57	1	0	0	0	2	10.94
69	0º to 30º	mild	5.6	98	5.1	0	0	31	88	1.76	1	0	0	0	12	-18.61
69	30º to 0º	mild	5.6	98	5.1	0	0	31	88	1.76	1	0	0	0	12	-3.29
70	0º to 30º	mild	9	93	5.7	0	0	54	98	1.85	1	0	0	0	15	4.36
70	30º to 0º	mild	9	93	5.7	0	0	54	98	1.85	1	0	0	0	15	-0.75
71	0º to 30º	mild	8	95	10.4	0	1	61	59.8	1.64	1	0	0	0	5	-8.73
71	30º to 0º	mild	8	95	10.4	0	1	61	59.8	1.64	1	0	0	0	5	13.22
73	0º to 30º	mild	10.5	96	11.1	0	1	60	64	1.5	0	0	0	1	15	-11.31

73	30º to 0º	mild	10.5	96	11.1	0	1	60	64	1.5	0	0	0	1	15	-5.93
76	0º to 30º	mild	7.6	97	0.2	0	0	36	65	1.71	0	0	0	0	16	-31.22
76	30º to 0º	mild	7.6	97	0.2	0	0	36	65	1.71	0	0	0	0	16	-10.08
79	0º to 30º	mild	9.4	96	NA	NA	0	62	91	1.81	0	0	0	0	15	-10.17
79	30º to 0º	mild	9.4	96	NA	NA	0	62	91	1.81	0	0	0	0	15	4.89
87	0º to 30º	mild	10	95	10	0	1	57	57	1.53	0	0	0	0	5	-23.86
87	30º to 0º	mild	10	95	10	0	1	57	57	1.53	0	0	0	0	5	4.6
43	0º to 30º	control	3.8	96	3.2	0	0	57	79.4	1.67	1	0	0	0	14	-28.77
43	30º to 0º	control	3.8	96	3.2	0	0	57	79.4	1.67	1	0	0	0	14	-15.01
72	0º to 30º	control	1	95	0	0	1	55	59	1.59	0	0	0	0	0	-19.26
72	30º to 0º	control	1	95	0	0	1	55	59	1.59	0	0	0	0	0	-2.77
74	0º to 30º	control	0	97	1	0	0	41	72	1.7	1	0	0	0	NA	-8.03
74	30º to 0º	control	0	97	1	0	0	41	72	1.7	1	0	0	0	NA	-2.02
80	0º to 30º	control	1	94	1	0	1	46	72	1.65	0	0	0	0	5	-7.85
80	30º to 0º	control	1	94	1	0	1	46	72	1.65	0	0	0	0	5	-11.13
81	0º to 30º	control	1.6	96	1	0	1	56	62	1.66	1	0	0	0	10	-38.19
81	30º to 0º	control	1.6	96	1	0	1	56	62	1.66	1	0	0	0	10	-16.06
82	0º to 30º	control	4	93	3.5	1	0	29	73	1.72	0	0	0	0	8	-17.66
82	30º to 0º	control	4	93	3.5	1	0	29	73	1.72	0	0	0	0	8	1.8
83	0º to 30º	control	2.3	95	2	0	1	57	84	1.52	1	0	0	0	6	-17.69
83	30º to 0º	control	2.3	95	2	0	1	57	84	1.52	1	0	0	0	6	-7.51
84	0º to 30º	control	4	96	0.2	0	1	53	58	1.53	0	0	0	0	9	-25.77
84	30º to 0º	control	4	96	0.2	0	1	53	58	1.53	0	0	0	0	9	-8.86
86	0º to 30º	control	1	94	1	0	1	52	59	1.57	0	0	0	0	0	-20.43
86	30º to 0º	control	1	94	1	0	1	52	59	1.57	0	0	0	0	0	-3.74
88	0º to 30º	control	3	95	3.4	0	0	55	108	1.82	1	0	0	0	8	2.79
88	30º to 0º	control	3	95	3.4	0	0	55	108	1.82	1	0	0	0	8	15.3

89	0º to 30º	control	1	95	0.8	0	0	40	71	1.74	0	0	0	0	7	-12.19
89	30º to 0º	control	1	95	0.8	0	0	40	71	1.74	0	0	0	0	7	12.13
91	0º to 30º	control	3.9	98	0	0	1	29	45	1.69	0	0	0	0	16	-21.68
91	30º to 0º	control	3.9	98	0	0	1	29	45	1.69	0	0	0	0	16	-3.91
92	0º to 30º	control	1	96	0	0	1	26	58	1.62	0	0	0	0	0	-17.1
92	30º to 0º	control	1	96	0	0	1	26	58	1.62	0	0	0	0	0	-9.32
93	0º to 30º	control	4	96	3	0	1	58	59	1.58	0	0	0	1	5	-9.76
93	30º to 0º	control	4	96	3	0	1	58	59	1.58	0	0	0	1	5	-10.6

AHI, apnea-hypopnea index; SpO_2 , arterial oxygen saturation by pulse oximetry; ODI4, 4% oxygen desaturation index; CT90, % of total sleep time with SpO_2 lower than 90%; AHT, arterial hypertension; DM, diabetes mellitus; DLP, dyslipidemia; rCBF, relative cerebral blood flow.
