# Supplementary material for: Cerebral vasoreactivity in response to a head-of-bed position change is altered in patients with moderate and severe obstructive sleep apnea
Source: PLoS One. 2018 Mar 14;13(3):e0194204. doi: 10.1371/journal.pone.0194204 (PMC5851619; doi:10.1371/journal.pone.0194204)
Supplement: S3 Table — Data shown as median (interquartile range) or number of cases (percentages). BMI, body mass index; AHT, arterial hypertension; AHI, apnea-hypopnea index; SpO2, arterial oxygen saturation; CT90, % of total sleep time with SpO2 lower than 90%; ODI4, 4% oxygen desaturation index; OSA, obstructive sleep apnea; CPAP, continuous positive airway pressure treatment. (PDF) [file pone.0194204.s003.pdf]

S3 Table: Pre-CPAP treatment a) demographics, clinical characteristics and b) sleep study results of the subgroup with severe OSA remeasured after two years of CPAP treatment, the subgroup with severe OSA measured one time, and all the group with severe OSA.

a)

	**Severe OSA measured one time (n=15)**	**Severe OSA remeasured (n=13)**	**p**	**Severe OSA total (n=28)**
**Age (y.)**	61 (53.5, 63)	57 (53, 63)	0.764	58 (53, 63.3)
**Males n (%)**	12 (43)	10 (36)	0.871	22 (79)
**BMI (kg/cm_2)**	32 (30, 38)	33 (31, 36)	0.964	33 (30, 36)
**AHT n (%)**	9 (32)	7 (25)	0.768	16 (57)
**Epworth**	11 (3,14)	14 (9, 15)	0.267	11.5 (7.5, 15)

b)

**AHI (n./hour)**	75 (43, 92)	73 (47, 80)	1	73.5 (47, 87)
**Mean SpO_2 (%)**	93 (92, 94)	92 (90, 94)	0.273	93 (91, 94)
**CT90 (%)**	14 (9, 26)	23 (11, 38)	0.279	16 (9, 27)
**ODI4 (%)**	55 (36, 76)	69 (46, 74)	0.525	63.5 (41, 75)

Data shown as median (interquartile range) or number of cases (percentages).
BMI, body mass index; AHT, arterial hypertension; AHI, apnea-hypopnea index; SpO_2, arterial oxygen saturation by pulse oximetry; CT90, % of total sleep time with SpO_2 lower than 90%; ODI4, 4% oxygen desaturation index; OSA, obstructive sleep apnea; CPAP, continuous positive airway pressure treatment.
