# Supplementary material for: Cerebral vasoreactivity in response to a head-of-bed position change is altered in patients with moderate and severe obstructive sleep apnea
Source: PLoS One. 2018 Mar 14;13(3):e0194204. doi: 10.1371/journal.pone.0194204 (PMC5851619; doi:10.1371/journal.pone.0194204)
Supplement: S4 Table — AHI, apnea-hypopnea index; SpO2, arterial oxygen saturation; ODI4, 4% oxygen desaturation index; CT90, % of total sleep time with SpO2 lower than 90%; CPAP, continuous positive airway pressure; AHT, arterial hypertension; DM, diabetes mellitus; DLP, dyslipidemia; rCBF, relative cerebral blood flow. (PDF) [file pone.0194204.s004.pdf]

S4 Table: Demographics, sleep study results, clinical characteristics, and optical study results of the severe obstructive sleep apnea patients measured before and after CPAP treatment.

**ID**	**Positional change**	**Male (yes=0)**	**Patient type**	**AHI (n./hour)**	**Mean SpO_2 (%)**	**ODI4 (%)**	**CT90 (%)**	**Smoking (current/ exsmoker=1)**	**Age (y.) pre-CPAP**	**Height (m)**	**Weight pre-CPAP (Kg)**	**Weight post-CPAP (Kg)**	**AHT pre-CPAP (yes=1)**	**AHT post-CPAP (yes=1)**	**DM (yes=1)**	**DLP (yes=1)**	**Time under treatment between measurements (y.)**	**Epworth pre-CPAP**	**Epworth post-CPAP**	**rCBF (%) pre-CPAP**	**rCBF (%) post-CPAP**
3	0º to 30º	0	severe	99.5	92	73.7	22.8	0	63	1.59	94.00	84.80	1	1	1	0	2.5	15	2	-24.64	-18.48
3	30º to 0º	0	severe	99.5	92	73.7	22.8	0	63	1.59	94.00	84.80	1	1	1	0	2.5	15	2	32.74	6.48
5	0º to 30º	1	severe	74.2	94	78.4	10.8	1	54	1.76	86.20	83.00	1	1	0	0	2.4	7	0	-21.3	-28.91
5	30º to 0º	1	severe	74.2	94	78.4	10.8	1	54	1.76	86.20	83.00	1	1	0	0	2.4	7	0	0.36	-0.36
6	0º to 30º	1	severe	72.8	89	69.8	38	0	73	1.61	90.00	94.00	1	1	0	0	2.4	6	0	4.93	-15.93
6	30º to 0º	1	severe	72.8	89	69.8	38	0	73	1.61	90.00	94.00	1	1	0	0	2.4	6	0	9.52	14.77
8	0º to 30º	1	severe	91.1	86	88	58.6	1	49	1.81	152.00	154.80	0	0	0	0	2.5	23	6	-10.59	-13.74
8	30º to 0º	1	severe	91.1	86	88	58.6	1	49	1.81	152.00	154.80	0	0	0	0	2.5	23	6	10.39	-3.81
9	0º to 30º	0	severe	96.2	90	85.3	41.7	0	47	1.6	79.40	78.80	0	0	0	0	2.4	20	10	-7.64	-1.51
9	30º to 0º	0	severe	96.2	90	85.3	41.7	0	47	1.6	79.40	78.80	0	0	0	0	2.4	20	10	11.85	11.72
11	0º to 30º	1	severe	79.8	92	66.2	23.1	1	68	1.73	100.00	98.40	0	1	1	1	2.3	9	6	-16.17	-16.67
11	30º to 0º	1	severe	79.8	92	66.2	23.1	1	68	1.73	100.00	98.40	0	1	1	1	2.3	9	6	12.47	4.54
13	0º to 30º	1	severe	78.1	93	72.4	16.9	1	58	1.81	114.00	112.20	1	1	0	0	2.3	9	4	-28.33	-30.13
13	30º to 0º	1	severe	78.1	93	72.4	16.9	1	58	1.81	114.00	112.20	1	1	0	0	2.3	9	4	6.71	-2.69
25	0º to 30º	1	severe	46.1	96	45.9	5.2	1	57	1.65	89.00	100.00	1	1	0	1	1.7	15	8	-16.25	-10.09
25	30º to 0º	1	severe	46.1	96	45.9	5.2	1	57	1.65	89.00	100.00	1	1	0	1	1.7	15	8	22.15	26.2
27	0º to 30º	1	severe	71.2	86	70.5	58.3	1	53	1.58	89.00	90.00	1	1	0	1	2.0	23	13	-2.85	-8.1
27	30º to 0º	1	severe	71.2	86	70.5	58.3	1	53	1.58	89.00	90.00	1	1	0	1	2.0	23	13	35.96	4.51
30	0º to 30º	1	severe	34.2	91	35.4	18.7	1	57	1.78	98.20	104.60	1	1	0	1	2.0	15	8	-15.64	-2.52
30	30º to 0º	1	severe	34.2	91	35.4	18.7	1	57	1.78	98.20	104.60	1	1	0	1	2.0	15	8	12.76	20.29
33	0º to 30º	1	severe	46.5	94	42.6	8.6	0	47	1.79	82.00	83.40	0	0	0	0	2.0	11	5	-21.09	-20.87
33	30º to 0º	1	severe	46.5	94	42.6	8.6	0	47	1.79	82.00	83.40	0	0	0	0	2.0	11	5	5.75	-5.75
45	0º to 30º	0	severe	33.8	95	19.3	0.8	0	67	1.6	66.00	67.20	0	0	0	1	1.8	6	6	-31.14	-32.84

45	30º to 0º	0	severe	33.8	95	19.3	0.8	0	67	1.6	66.00	67.20	0	0	0	1	1.8	6	6	-5.83	-11.64
47	0º to 30º	1	severe	71.5	91	64	31.4	1	62	1.66	118.00	115.00	1	1	1	1	1.7	14	4	-1.73	14.62
47	30º to 0º	1	severe	71.5	91	64	31.4	1	62	1.66	118.00	115.00	1	1	1	1	1.7	14	4	11.06	11.08

AHI, apnea-hypopnea index; SpO_2 , arterial oxygen saturation by pulse oximetry; ODI4, 4% oxygen desaturation index; CT90, % of total sleep time with SpO_2 lower than 90%; CPAP, continuous positive airway pressure; AHT, arterial hypertension; DM, diabetes mellitus; DLP, dyslipidemia; rCBF, relative cerebral blood flow.
